# Twenty-Year Experience with Surgery for Native and Prosthetic Mitral Valve Endocarditis

**DOI:** 10.3390/medicina59061060

**Published:** 2023-05-31

**Authors:** Antonella Galeone, Jacopo Gardellini, Venanzio Di Nicola, Fabiola Perrone, Vincenzo Boschetti, Renato Di Gaetano, Francesco Onorati, Giovanni Battista Luciani

**Affiliations:** 1 Department of Surgery, Dentistry, Pediatrics and Gynecology, Division of Cardiac Surgery, University of Verona, 37126 Verona, Italy; 2 Department of Cardiology, Azienda Sanitaria dell’Alto Adige, 39100 Bolzano, Italy; renato.digaetano@sabes.it

**Keywords:** mitral valve, infective endocarditis, prosthetic valve endocarditis

## Abstract

*Background and Objectives*: To evaluate the early and long-term results of surgical treatment of isolated mitral native and prosthetic valve infective endocarditis. *Materials and Methods*: All patients undergoing mitral valve repair or replacement for infective endocarditis at our institution between January 2001 and December 2021 were included in the study. The preoperative and postoperative characteristics and mortality of patients were retrospectively reviewed. *Results:* A total of 130 patients, 85 males and 45 females, with a median age of 61 ± 14 years, underwent surgery for isolated mitral valve endocarditis during the study period. There were 111 (85%) native and 19 (15%) prosthetic valve endocarditis cases. Fifty-one (39%) patients died during the follow-up, and the overall mean patient survival time was 11.8 ± 0.9 years. The mean survival time was better in patients with mitral native valve endocarditis compared to patients with prosthetic valve endocarditis (12.3 ± 0.9 years vs. 8 ± 1.4 years; *p* = 0.1), but the difference was not statistically significant. Patients who underwent mitral valve repair had a better survival rate compared to patients who had mitral valve replacement (14.8 ± 1.6 vs. 11.3 ± 1 years; *p* = 0.06); however, the difference was not statistically significant. Patients who underwent mitral valve replacement with a mechanical prosthesis had a significantly better survival rate compared to patients who received a biological prosthesis (15.6 ± 1.6 vs. 8.2 ± 0.8 years; *p* < 0.001). Patients aged ≤60 years had significantly better survival compared to patients aged >60 years (17.1 ± 1.1 vs. 8.2 ± 0.9; *p* < 0.001). Multivariate analysis showed that the patient’s age >60 years at the time of surgery was an independent risk factor for mortality, while mitral valve repair was a protective factor. Eight (7%) patients required reintervention. Freedom from reintervention was significantly higher in patients with mitral native valve endocarditis compared to patients with prosthetic valve endocarditis (19.3 ± 0.5 vs. 11.5 ± 1.7 years; *p* = 0.04). *Conclusions*: Surgery for mitral valve endocarditis is associated with considerable morbidity and mortality. The patient’s age at the time of surgery represents an independent risk factor for mortality. Mitral valve repair should be the preferred choice whenever possible in suitable patients affected by infective endocarditis.

## 1. Introduction

Infective endocarditis is a challenging and life-threatening disease with an estimated incidence of 3–10 cases per 100,000 people per year [1]. Despite diagnostic and therapeutic advances, the prognosis remains poor, with a 14–22% in-hospital mortality rate and up to 50% mortality at 10 years [2]. Surgery is required in 25–50% of cases in the acute phase and in 20% to 40% of cases during convalescence. Previous reports showed that survival after surgery for mitral valve infective endocarditis is worse than after surgery for aortic valve infective endocarditis [3,4,5]. Prosthetic valve endocarditis occurs in 1% to 6% of patients with previous heart valve replacement and accounts for more than 20% of all infective endocarditis [6]. The complication rate is significantly higher for mitral prosthetic valve endocarditis compared to mitral native valve endocarditis [7]. Previous studies suggest better outcomes when the mitral valve is repaired rather than replaced, with a lower reinfection and reoperation rate [8,9,10]. In this study, we reviewed our single-center experience of surgical treatment of both isolated mitral native and prosthetic valve endocarditis over a 20-year period and reported the early and long-term results of surgery for mitral valve infective endocarditis with either mitral valve repair or replacement. For native mitral valve endocarditis, the decision to repair or replace the mitral valve depended on the surgeon’s personal choice, the extension of the disease, and the possibility of ensuring the complete eradication of the infection.

## 2. Materials and Methods

The study was conducted in accordance with the Declaration of Helsinki and approved by the Ethics Committee of the Azienda Ospedaliera Universitaria Integrata of Verona (approval number: 64927; date of approval: 30 November 2020). Written informed consent was waived by the Ethics Committee.

All consecutive adult patients undergoing surgery for native or prosthetic mitral valve endocarditis at our institution between January 2001 and December 2021 were included in the study. Patients’ characteristics, perioperative data, and in-hospital outcomes were extracted from patients’ paper-based and electronic medical records. The diagnosis of infective endocarditis was based on the revised Duke’s criteria [11]. Patients were scheduled for surgery according to current guidelines [12]. Infective endocarditis can be classified as acute or healed based on the severity of the clinical presentation and the progression of the disease. Acute endocarditis has generally been defined as endocarditis during the 6-week antibiotic treatment, while healed endocarditis refers to endocarditis after the 6-week antibiotic treatment [13].

All operations were performed through a median full sternotomy, standard cardiopulmonary bypass, and cold blood or crystalloid cardioplegia. During the study period, mitral native valve endocarditis was managed either by mitral valve repair or replacement with a biological or mechanical prosthesis based on the surgeon’s personal choice, the extension of the disease, and the completeness of the eradication of the infection; prosthetic valve endocarditis was managed with mitral valve replacement.

Follow-up data were collected until March 2023 via phone and e-mail contact with patients, family members, family physicians, and cardiologists. Subsequent hospitalization and routine visit data were collected from hospital records and cardiology reports. The follow-up time was calculated either to death or to the last verified contact with the patient. Clinical outcomes of interest included mortality and reintervention for bioprosthetic valve dysfunction. Mortality was defined according to Valve Academic Research Consortium 3 as: periprocedural (occurring ≤30 days after the index procedure or >30 days but during the index hospitalization), early (occurring >30 days but ≤1 year after the index hospitalization), and late mortality (occurring >1 year after the index hospitalization) [13]. Bioprosthetic valve dysfunction was defined as the presence of structural valve dysfunction, non-structural valve dysfunction, infective endocarditis, and thrombosis [14].

Categorical variables are expressed as numbers and percentages and compared with χ^2^ test. Continuous variables with a skewed distribution are presented as the median and interquartile range and compared with the Mann–Whitney U test. The Kaplan–Meier method was used to draw survival curves, and the log-rank test was used to compare survival among groups. The reverse Kaplan–Meier survival curve was used to calculate the follow-up rate. The completeness of follow-up was calculated according to Clark’s formula [15]. Hazard ratios for mortality were determined by univariate and multivariate Cox proportional hazards regression analysis, with the data presented as a hazard ratio with 95% Cis. A two-tailed *p* value < 0.05 was taken to indicate statistical significance. Statistical analysis was performed using Sigmaplot version 12.0 (Systat Software Inc., San Jose, CA, USA).

## 3. Results

### 3.1. Demography

During the study period, 462 patients underwent surgery for native (*n* = 309, 67%) and prosthetic (*n* = 153, 33%) valve infective endocarditis at our institution. Among them, 130 (28%) patients, 85 males and 45 females, with a median age of 61 ± 14 years, required surgery for isolated mitral valve endocarditis. There were 111 (85%) native valve and 19 (15%) prosthetic valve endocarditis cases. Pre-, intra-, and perioperative characteristics of the whole population and according to the type of infective endocarditis are listed in Table 1. *Streptococcus* spp. were the most common cause of mitral native valve endocarditis, while *coagulase-negative staphylococci* were most commonly responsible for prosthetic valve endocarditis.

Mitral valve repair was performed in 28 (22%) patients with mitral native valve endocarditis. Patients with mitral prosthetic valve endocarditis were more frequently female, significantly older (71 [69–74] vs. 61 [49–71] years; *p* < 0.001), underwent more frequent mitral valve replacement with a biological prosthesis, and had longer cardiopulmonary bypass and aortic cross clamping times compared to patients with mitral native valve endocarditis. In patients with native mitral valve endocarditis, no difference was found in pre- and perioperative characteristics between patients who underwent mitral valve repair and patients who underwent mitral valve replacement (Table 2).

### 3.2. Survival

Four patients were lost at follow-up, and the completeness of follow-up was 95.6% according to Clark’s formula [15]. The mean follow-up duration was 9.3 ± 0.6 years (median: 7.7 [4.7–13.7]).

**Figure 1:**
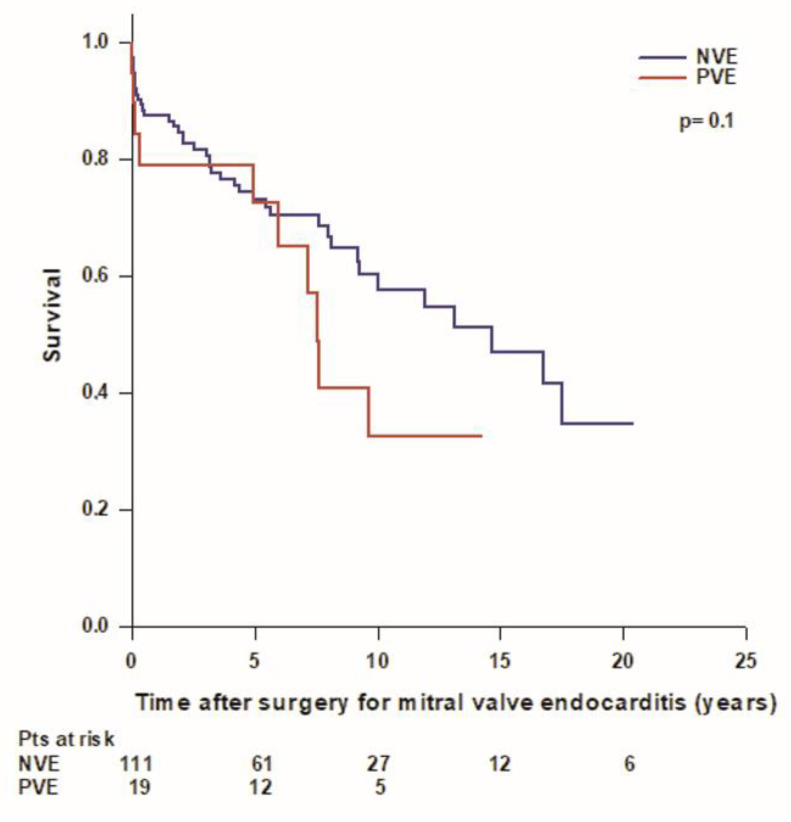
Survival after surgery for mitral valve endocarditis in patients with mitral native (NVE, blue line) and prosthetic valve endocarditis (PVE, red line).

**Figure 2:**
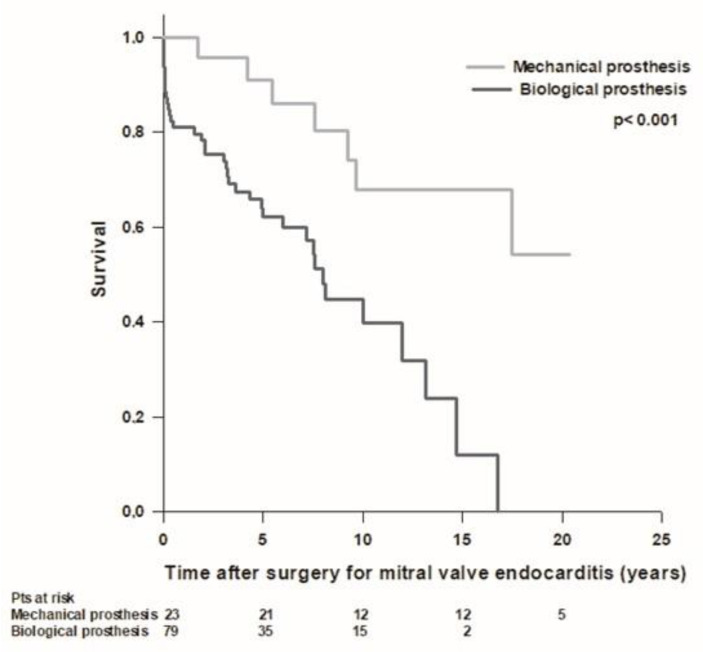
Patient survival after surgery for mitral valve replacement for endocarditis with mechanical (light grey line) and biological (dark gray line) mitral valve prostheses.

**Figure 3:**
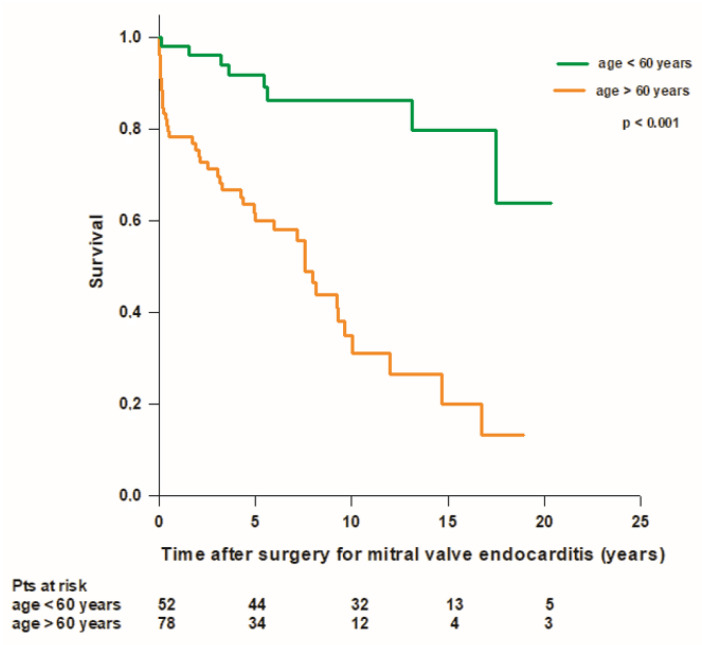
Survival after surgery for mitral valve endocarditis in patients with less than 60 years (green line) and more than 60 years (orange line) at the time of surgery.

**Figure 4:**
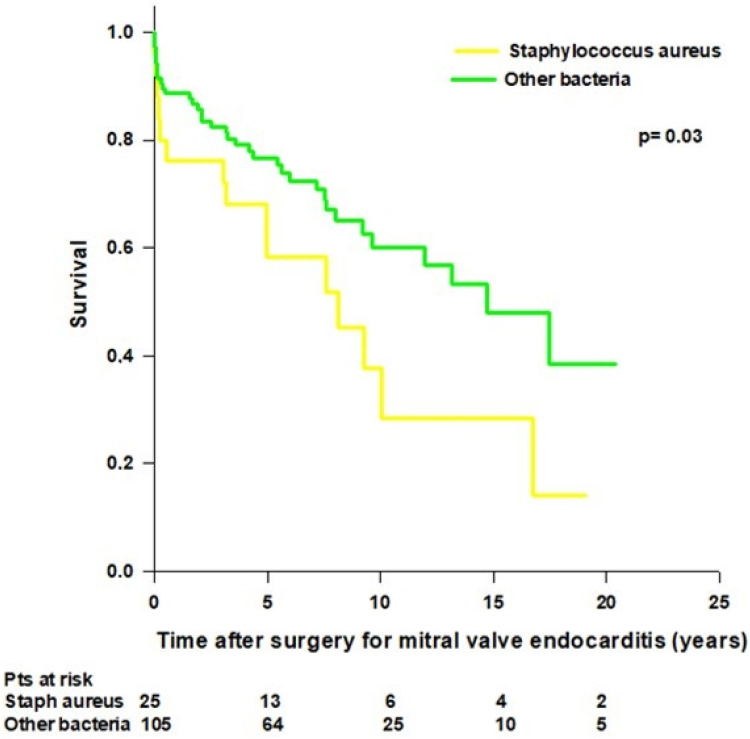
Patient survival after surgery for mitral valve endocarditis caused by *Staphylococcus aureus* (yellow line) and other bacteria (green line).

We recorded a total of 51 (39%) deaths, including 13 (10%) periprocedural deaths, 5 (4%) early deaths, and 33 (25%) late deaths. Overall mean patient survival time was 11.8 ± 0.9 years, and long-term survival rates were 94.6%, 86.1%, 72.9%, 55.1%, 43.7%, and 32.3% at 30 days, 1, 5, 10, 15, and 20 years, respectively. Forty-one (37%) patients with mitral native valve endocarditis and 10 (53%) patients with mitral prosthetic valve endocarditis died during the follow-up. In the mitral native valve endocarditis group, we recorded 10 (9%) periprocedural deaths, 4 (4%) early deaths, and 27 (24%) late deaths, while in the mitral prosthetic valve endocarditis group, we recorded 3 (16%) periprocedural deaths, 1 (5%) early death, and 6 (32%) late deaths. The mean survival time was better in patients with mitral native valve endocarditis compared to patients with mitral prosthetic valve endocarditis (12.3 ± 0.9 years vs. 8 ± 1.4 years; *p* = 0.1), but the difference was not statistically significant (Figure 1). In patients operated on for mitral native valve endocarditis, patients who underwent mitral valve repair had a better survival rate compared to patients who underwent mitral valve replacement, but the difference was not statistically significant (mean survival time 14.8 ± 1.6 vs. 11.3 ± 1 years; *p* = 0.06). Patients with both mitral native and prosthetic valve endocarditis who underwent mitral valve replacement with a mechanical prosthesis had significantly better survival compared to patients who received a biological prosthesis (mean survival time 15.6 ± 1.6 vs. 8.2 ± 0.8 years; *p* < 0.001) (Figure 2). Patients aged ≤60 years had significantly better survival compared to patients aged >60 years (17.1 ± 1.1 vs. 8.2 ± 0.9; *p* < 0.001) (Figure 3). Patients with mitral native or prosthetic infective endocarditis caused by *Staphylococcus aureus* had significantly worse survival compared to patients with endocarditis caused by other bacteria (mean survival time: 8.5 ± 1.6 vs. 12.7 ± 0.9 years; *p* = 0.03) (Figure 4). The mean survival time was lower in females compared to males (9.3 ± 1.4 vs. 12.9 ± 1 years; *p* = 0.08), but the difference was not statistically significant. Patients with healed infective endocarditis had significantly better survival compared to patients with active infective endocarditis (mean survival time: 14.2 ± 1.3 vs. 9.8 ± 1 year; *p* = 0.02). No difference was found in mean survival time between patients operated on in the early period of our experience (2001–2010, *n* = 43) and patients operated on in the last decade (2011–2021, *n* = 87) (mean survival time: 11.3 ± 1.2 years vs. 8.9 ± 0.6 years; *p* = 0.4).

A univariate analysis was performed with preoperative and perioperative variables. At univariate analysis, patients’ age >60 years at time of surgery and *Staphylococcus aureus* infection were independent risk factors for mortality, while mitral valve repair and mitral valve replacement with a mechanical prosthesis were protective factors. Significant variables from the univariate analysis were entered in the Cox multivariate regression. At multivariate analysis, only the patient’s age >60 years at the time of surgery was an independent risk factor for mortality, and mitral valve repair was a protective factor (Table 3).

### 3.3. Reoperations

**Figure 5:**
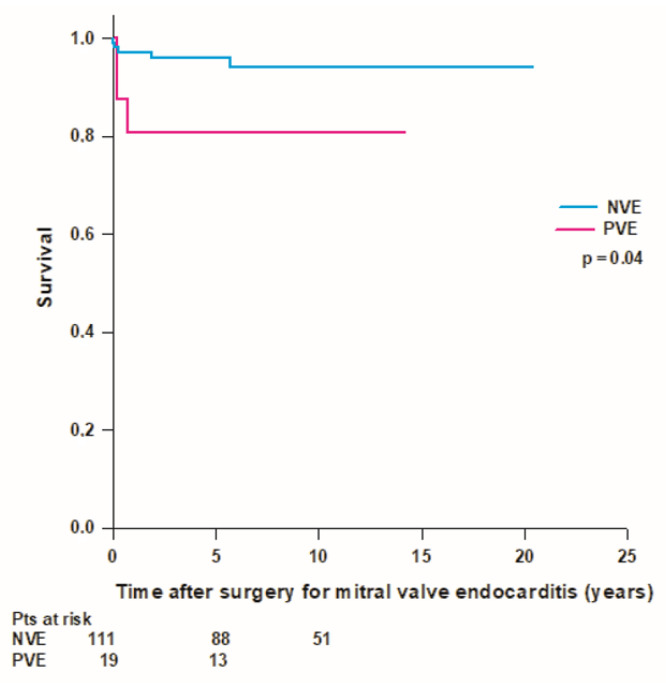
Freedom from reoperation after surgery for mitral valve endocarditis in patients with mitral native (NVE, pink line) and prosthetic (PVE, light blue line) valve endocarditis.

Eight patients (7%), 5 (5%) with mitral native valve endocarditis, and 3 (16%) with mitral prosthetic valve endocarditis required reintervention at a mean time of 1.1 ± 1.9 years (median time: 0.2 [0.1–1.3] years) after surgery for mitral valve infective endocarditis. Five patients (4%), 2 (2%) with mitral native valve endocarditis, and 3 (16%) with mitral prosthetic valve endocarditis required reintervention for recurrent infective endocarditis. Three (2%) patients with mitral native valve endocarditis required reoperation for the following non-infectious indications: failure of prior mitral valve repair, severe aortic regurgitation, and periprosthetic leak of the aortic valve prosthesis implanted for aortic stenosis at the time of surgery for infective endocarditis. The overall mean survival free from reintervention was 18.9 ± 0.5 years. Patients with mitral native valve endocarditis had a significantly higher mean survival time free from reoperation compared to patients with mitral prosthetic valve endocarditis (19.3 ± 0.5 vs. 11.5 ± 1.7 years; *p* = 0.04) (Figure 5).

## 4. Discussion

Infectious endocarditis represents a huge burden worldwide. Both incidence and mortality of infective endocarditis have increased sharply during the past 30 years and show an upward temporal trend annually [16]. A report analyzing the results of 4195 cases of infective endocarditis from 13 European countries showed that native valve endocarditis decreased and prosthetic valve and device-related endocarditis both increased in recent years [17]. Surgical treatment also increased, and in-hospital and 6-month mortality rates significantly decreased in recent years [17]. In our study, the total number of patients who were diagnosed with isolated mitral valve endocarditis and required surgical treatment doubled over the last two decades; however, despite improvements in diagnostic and therapeutic procedures, mortality after surgery remained unchanged. We found periprocedural mortality in the whole population of 10%, which is comparable to that of previously published studies ranging from 3.8% to 15.4% [10,18,19,20,21,22]. Long-term survival rates were 73% at 5 years and 55% at 10 years, and both are similar to those of most recent studies [10,19,20,21]. Some authors showed better survival in patients operated on for mitral native valve endocarditis compared to patients operated on for mitral prosthetic valve endocarditis [23], while others didn’t find any difference in survival between native and prosthetic valve endocarditis [5]. In the present experience, mean survival in patients with mitral native valve endocarditis showed a clinically relevant difference compared to mean survival in patients with mitral prosthetic valve endocarditis, with a difference of more than 4 years. However, the difference did not prove statistically significant, possibly due to the sample size. Similar to previous studies [5], we also found that patients with healed infective endocarditis at the time of surgery had significantly better outcomes with a 10-year survival rate of 69% compared to patients with active infective endocarditis, which had a 10-year survival rate of 44%.

In our series, the median age of the whole population was 64 years, which is slightly higher compared to previously published studies reporting a median age of 60 years [4,5,19]. Patient age at the time of surgery was the only independent risk factor for mortality, supporting the results of the study of Perrotta and coll. [10], while mitral valve repair was a protective factor. Since the first reported case of successful mitral valve repair for infective endocarditis by Dreyfus in 1990 [24], several authors have suggested that in patients undergoing surgery for endocarditis, mitral valve repair may be safely performed and is often associated with a better outcome. In a multicentric study including 1970 patients undergoing isolated primary mitral valve repair or replacement for active infective endocarditis between 1998 and 2010 in North America, the rate of mitral valve repair in infective endocarditis increased from 10.7% to 19.4% over the study period [9]. Our proportion of mitral valve repair for native valve endocarditis was 22%, which is at the lower end of what has recently been reported, ranging from 27% to 80% [10,18,19,21]. Data in the literature strongly suggest that mitral valve repair is a good option in mitral native valve endocarditis, although, in our series, we failed to demonstrate better survival in patients with native valve endocarditis who underwent mitral valve repair. Similarly, Defauw and coll. [20] did not find a survival benefit of mitral valve repair during the entire follow-up period; however, when a landmark analysis was performed, improved patient survival beyond 1 year after surgery was seen in patients who underwent valve repair compared to patients who underwent valve replacement. A systemic review including 470 patients who underwent mitral valve repair and 724 patients who underwent mitral valve replacement for mitral valve endocarditis showed that mitral valve repair is associated with lower in-hospital mortality (2.3% vs. 14.4%) and long-term mortality (7.8% vs. 40.5%) and lower rates of reoperation, recurrent endocarditis, and cerebrovascular events [18]. Since this review, those findings have been supported by various published results [25,26]. A meta-analysis conducted on 17 publications with a total population of 3759 patients, with 1180 patients having undergone mitral valve repair and 2579 patients having undergone valve replacement, showed that patients who underwent mitral valve repair may benefit from a lower risk of early mortality, a higher long-term survival rate, and a lower risk of recurrent endocarditis [27]. However, a similar risk of reoperation was observed for both groups [27]. A more recent meta-analysis involving 23 articles published from 2000 to 2021 and including 25,615 patients, 10,719 of whom received mitral valve repair and 14,896 received mitral valve replacement from 1980 to 2017, showed fewer adverse events and early or long-term mortality in patients with mitral valve repair [28]. However, more reoperations existed in this patient group, and the reinfection rate was similar between the two groups [28].

The advantages of mitral valve repair over valve replacement are based on better preservation of left ventricular function and a reduced rate of prosthetic valve-related complications. Potential concerns in mitral valve repair are durability, the possibility of recurrent infection due to incomplete resection of infected valvular tissue, and the use of prosthetic annuloplasty rings [29]. The choice of mitral valve repair versus replacement depends on pathology and the underlying patient’s conditions, and the effect on outcomes is still controversial in recent literature. Mitral valve replacement is required for the most extensive pathology, including mitral annular invasion and calcification and tissue and cusp destruction. In patients with more comorbidities and extensive valve destruction, extended repair has worse outcomes, similar to valve replacement [21]. Both mechanical and bioprosthetic valves have been used in mitral valve replacement, with similar survival rates and freedom from reinfection. Greason et al. showed no significant difference between mechanical and bioprosthetic valves in overall mortality or recurrence of infection over a 35-year follow-up [30]. The risk of reoperation, however, appears to be higher among patients with tissue valve replacement; thus, valve choice should be individualized according to age, life expectancy, and presence of comorbidities [29]. Recent reports suggest that the use of a mechanical mitral valve prosthesis is associated with lower long-term mortality compared to the biological prosthesis in non-elderly native mitral valve infective endocarditis patients [22]. In our series, patients with both isolated mitral native and prosthetic valve endocarditis who underwent mitral valve replacement with a mechanical prosthesis had significantly better survival compared to patients who received a biological prosthesis. This observation, however, may be partly biased by the younger average age at surgery of patients receiving mechanical prostheses.

In our experience, reoperation rates for recurrent infective endocarditis were lower than those previously published [10], and patients with mitral prosthetic valve endocarditis had a lower freedom from reintervention and experienced more recurrence of endocarditis compared to patients with mitral native valve endocarditis. These results are consistent with those of Moore and colleagues [21], who reported higher reinfection rates after mitral prosthetic valve endocarditis.

Previous studies demonstrated a shift in the microbiology of infective endocarditis, with *Staphylococcus aureus* being the leading cause of endocarditis worldwide [31]. This shift could be attributed to growing risk factors for *Staphyloccoccus aureus*-associated infective endocarditis such as injection drug abuse, invasive procedures, and health care exposure. Indeed, infective endocarditis has become a disease in which the presentation is more acute than previously described and potentially lethal due to the growing antimicrobial resistance of *Staphyloccoccus aureus.* In contrast with these reports, in our series, *Staphyloccoccus aureus* was not the most common cause of infective endocarditis. Similarly, Feringa [18] and Defauw [20] found that microorganisms from the *Streptococcus* group were the most common causative microorganisms identified in mitral valve endocarditis. The prognosis of *Staphyloccoccus *aureus** infective endocarditis remains poor, especially in the presence of congestive heart failure, severe sepsis, major neurologic events, and prosthetic valve endocarditis [32]. Early surgery is independently associated with reduced overall mortality [32]. Accordingly, in our series, patients with *Staphylococcus aureus* infective endocarditis had a worse prognosis compared to patients with endocarditis caused by other bacteria; however, *Staphylococcus aureus* infection was an independent risk factor for mortality only at univariate analysis.

This study has some limitations. Since this is a single-center retrospective study covering 20 years, a risk of selection bias is unavoidable. Most likely, diagnostic approaches, medical treatment, surgical techniques, and postoperative management have evolved over the last two decades. Therefore, the results should be interpreted with caution.

## 5. Conclusions

Infective endocarditis is a challenging clinical condition. Despite progress in diagnostic and therapeutic management, surgery for isolated native and prosthetic mitral valve endocarditis is associated with considerable morbidity and mortality. The patient’s age at the time of surgery represents an independent risk factor for mortality. In the last twenty years, mitral valve repair has been shown to be a valuable alternative to mitral valve replacement in the setting of mitral valve infective endocarditis, as it is associated with decreased in-hospital and long-term mortality when compared to valve replacement. Mitral valve repair should be the preferred choice whenever possible in suitable patients affected by infective endocarditis.

## Figures and Tables

**Table 1:** Preoperative and perioperative characteristics of patients with isolated mitral native (m-NVE) and prosthetic valve endocarditis (m-PVE).

	All (*n* = 130)	m-NVE (*n* = 111)	m-PVE (*n* = 19)	*p*
Preoperative characteristic				
Male, *n* (%)	85 (65%)	77 (69%)	8 (42%)	0.04
Age, years	64 [51–72]	61 [49–71]	71 [69–74]	<0.001
BMI	23 [21–26]	23 [21–26]	24 [21–26]	0.5
BSA	1.8 [1.7–1.9]	1.8 [1.7–1.9]	1.7 [1.5–1.8]	0.01
Active IE	84 (65%)	69 (62%)	15 (79%)	0.2
Healed IE	46 (35%)	42(38%)	4 (21%)	0.2
Pre-operative cardiogenic shock	9 (7%)	8 (8%)	1 (5%)	0.8
Redo surgery	27	8 (7%)	19 (100%)	-
Microorganism				
GRAM+	80 (62%)	70 (63%)	10 (53%)	0.5
*Staphylococcus aureus*	25 (19%)	24 (22%)	1 (5%)	0.1
*Coagulase negative staphylococcus*	11 (8%)	7 (6%)	4 (21%)	0.09
*Streptococcus* spp.	31 (24%)	29 (26%)	2 (11%)	0.2
*Enterococcus* spp.	9 (7%)	8 (7%)	1 (5%)	0.8
Other GRAM+	4 (3%)	2 (2%)	2 (11%)	0.1
GRAM^−^	4 (3%)	2 (2%)	2 (11%)	0.1
Fungi	2 (2%)	0	2 (11%)	-
Culture negative infective endocarditis	26 (20%)	24 (22%)	2 (11%)	0.4
Unknown	18 (14%)	15 (14%)	3 (16%)	0.8
Intraoperative findings				
Vegetations	84 (65%)	72 (65%)	12 (63%)	0.9
Native valve perforation	52 (40%)	52 (47%)	0	-
Annular abscess	9 (7%)	6 (5%)	3 (16%)	0.2
Prosthetic valve dehiscence	10 (8%)	0	10 (53%)	-
Prosthetic valve perforation	4 (3%)	0	4 (21%)	-
Surgical technique				
Biological MVR	79 (61%)	62 (56%)	17 (89%)	0.01
Mechanical MVR	23 (18%)	21 (19%)	2 (11%)	0.5
MV repair	28 (22%)	28 (25%)	0	-
Concomitant procedure				
Biological AVR	3 (2%)	2 (2%)	1 (5%)	0.9
TV repair	1 (1%)	1 (1%)	0	-
CABG	12 (9%)	9 (8%)	3 (16%)	0.4
AAR	1 (1%)	1 (1%)	0	-
Perioperative characteristic				
CPB, min	106 [90–130]	100 [89–121]	136 [123–160]	<0.001
Aortic cross-clamping, min	82 [69–99]	79 [67–95]	100 [92–120]	<0.001
IABP	7 (5%)	5 (5%)	2 (11%)	0.6
ECMO	3 (2%)	3 (3%)	-	-
Re-exploration for bleeding	7 (5%)	6 (6%)	1 (5%)	0.9
CVA	1 (1%)	1 (1%)	0	-
PM implantation	3 (2%)	3 (3%)	1 (5%)	0.8
CRRT	7 (5%)	5 (5%)	2 (11%)	0.6

m-NVE—mitral native valve endocarditis; m-PVE—mitral prosthetic valve endocarditis; BMI—body mass index; BSA—body surface area; MVR—mitral valve replacement; AVR—aortic valve replacement; TV—tricuspid valve; CABG—coronary artery bypass-grafting; AAR—ascending aorta replacement; CPB—cardio-pulmonary bypass; IABP—intra-aortic balloon pump; ECMO—extra-corporeal membrane oxygenation; CVA—cerebrovascular accident; PM—pacemaker; CRRT—continuous renal replacement therapy.

**Table 2:** Preoperative and perioperative characteristics of patients with isolated mitral native c-valve endocarditis undergoing mitral valve repair or replacement.

	m-NVE (*n* = 111)	MV Repair (*n* = 28)	MVR (*n* = 83)	*p*
Preoperative characteristic				
Male, *n* (%)	77 (69%)	22 (79%)	55 (66%)	0.3
Age, years	61 [49–71]	59 [43–67]	64 [50–71]	0.1
BMI	23 [21–26]	23 [21–24]	24 [21–27]	0.1
BSA	1.8 [1.7–1.9]	1.8 [1.7–1.9]	1.8 [1.7–1.9]	0.4
Active IE	69 (62%)	14 (50%)	55 (66%)	0.1
Healed IE	42(38%)	14 (50%)	28 (34%)	0.1
Pre-operative cardiogenic shock	8 (8%)	2 (7%)	6 (7%)	0.6
Redo surgery	8 (7%)	0 (0%)	8 (10%)	-
Microorganism				
GRAM+	69 (62%)	16 (57%)	54 (65%)	0.7
*Staphylococcus aureus*	24 (22%)	5 (18%)	19 (23%)	0.7
*Coagulase negative staphylococcus*	7 (6%)	0 (0%)	7 (8%)	-
*Streptococcus* spp	28 (25%)	9 (32%)	20 (24%)	0.7
*Enterococcus* spp	8 (7%)	1 (4%)	7 (8%)	0.8
Other GRAM+	2 (2%)	1 (4%)	1 (1%)	0.8
GRAM^−^	2 (2%)	1 (4%)	1 (1%)	0.8
Fungi	0 (0%)	0 (0%)	0 (0%)	-
Culture negative infective endocarditis	24 (22%)	8 (29%)	16 (19%)	0.5
Unknown	15 (14%)	3 (11%)	12 (14%)	0.8
Intraoperative findings				
Vegetations	72 (65%)	16 (57%)	56 (63%)	0.7
Native valve perforation	52 (47%)	10 (36%)	42 (51%)	0.2
Annular abscess	6 (5%)	1 (4%)	5 (6%)	0.8
Surgical technique				
Biological MVR	62 (56%)	0 (0%)	62 (75%)	-
Mechanical MVR	21 (19%)	0 (0%)	21 (25%)	-
MV repair	28 (25%)	28 (100%)	0 (0%)	-
Concomitant procedure				
Biological AVR	2 (2%)	0 (0%)	2 (2%)	-
TV repair	1 (1%)	0 (0%)	1 (1%)	-
CABG	9 (8%)	1 (4%)	8 (10%)	0.7
AAR	1 (1%)	1 (4%)	0 (0%)	-
Perioperative characteristic				
CPB, min	100 [89–121]	104 [91–117]	99 [89–124]	0.9
Aortic cross-clamping, min	79 [67–95]	84 [68–99]	79 [67–93]	0.6
IABP	5 (5%)	0 (0%)	5 (6%)	-
ECMO	3 (3%)	0 (0%)	3 (4%)	-
Re-exploration for bleeding	6 (6%)	1 (4%)	5 (6%)	0.9
CVA	1 (1%)	1 (4%)	0 (0%)	-
PM implantation	3 (3%)	0 (0%)	3 (4%)	-
CRRT	5 (5%)	1 (4%)	4 (5%)	0.8

m-NVE—mitral native valve endocarditis; BMI—body mass index; BSA—body surface area; MVR—mitral valve replacement; AVR—aortic valve replacement; TV—tricuspid valve; CABG—coronary artery bypass-grafting; AAR—ascending aorta replacement; CPB—cardio-pulmonary bypass; IABP—intra-aortic balloon pump; ECMO—extra-corporeal membrane oxygenation; CVA—cerebrovascular accident; PM—pacemaker; CRRT—continuous renal replacement therapy.

**Table 3:** Predictors of mortality at univariate and multivariate analysis.

	Univariate Analysis		Multivariate Analysis	
	Hazard Ratio (95%CI)	*p*	Hazard Ratio (95%CI)	*p*
Patient’s age >60 years	5.64 (2.62–12.1)	<0.001	5.46 (2.42–11.8)	<0.001
Female sex	1.64 (0.93–2.88)	0.08		
Healed IE	0.8 (0.46–1.48)	0.5		
PVE	1.58 (0.79–3.19)	0.1		
*Staphylococcus aureus* infection	1.88 (1.02–3.44)	0.04	1.72 (1.01–2.98)	0.13
MV repair	0.42 (0.18–0.99)	0.04	0.38 (0.15–0.91)	0.03
Mechanical prosthesis	0.36 (0.15–0.84)	0.01	0.67 (0.25–1.76)	0.4
Postoperative IABP or ECMO	2.08 (0.74–5.8)	0.1		
Reintervention	1.32 (0.47–3.68)	0.5		

IE—infective endocarditis; PVE—prosthetic valve endocarditis; MV—mitral valve; IABP—intra-aortic balloon pump; ECMO—extra-corporeal membrane oxygenation.

## Data Availability

The data presented in this study are available upon request from the corresponding author.
